# HPV Vaccination Knowledge and Awareness Among Male University Students in Malaysia: A Cross-Sectional Study

**DOI:** 10.3390/vaccines14020126

**Published:** 2026-01-27

**Authors:** Siqi Li, Fatimah Ahmad Fauzi, Zhihai Jin, Rosliza Abdul Manaf

**Affiliations:** Department of Community Health, Faculty of Medicine & Health Sciences, Universiti Putra Malaysia, Seri Kembangan 43400, Malaysia; gs66650@student.upm.edu.my (S.L.); fatimah_fauzi@upm.edu.my (F.A.F.); gs65813@student.upm.edu.my (Z.J.)

**Keywords:** human papillomavirus, male students, vaccination, awareness, HPV vaccine

## Abstract

**Background/Objectives**: Human papillomavirus (HPV) vaccines are effective in preventing HPV infection and HPV-related cancers in both males and females. As sexual behavior plays a central role in HPV transmission, male vaccination is important not only for reducing HPV-associated diseases among men but also for limiting viral transmission at the population level. **Methods**: A cross-sectional study was conducted among male university students in Selangor, Malaysia. Data were collected using a structured questionnaire assessing sociodemographic characteristics, history of sexual intercourse, HPV-related knowledge, and awareness of HPV vaccination. Multivariable logistic regression analysis was performed to identify factors associated with HPV vaccination awareness. **Results**: Overall, 43.4% of the respondents demonstrated good awareness of HPV vaccination. Multivariable logistic regression analysis identified several factors significantly associated with awareness. Non-Muslim students were more likely to report good awareness of HPV vaccination than Muslim students (AOR = 2.724, 95% CI: 1.150–6.454, *p* < 0.001). Students who were in a relationship or married demonstrated higher awareness compared with single students (AOR = 3.830, 95% CI: 2.071–7.082, *p* < 0.001). HPV-related knowledge showed the strongest association, with participants possessing good knowledge being more likely to be aware of HPV vaccination (AOR = 7.012, 95% CI: 4.077–12.059, *p* < 0.001). In contrast, history of sexual intercourse was not significantly associated with HPV vaccination awareness after adjustment (*p* = 0.097). **Conclusions**: Awareness of HPV vaccination among male university students was influenced by religion, relationship status, and HPV-related knowledge. These findings highlight the need for targeted, male-inclusive vaccination education strategies that address sociodemographic differences. University-based interventions may play an important role in improving awareness and increasing HPV vaccine uptake in this population.

## 1. Introduction

Human papillomavirus (HPV) is one of the most prevalent sexually transmitted infections globally, with a high lifetime risk of acquisition among both women and men [1,2]. Although the majority of HPV infections are asymptomatic and self-limiting, persistent infection with oncogenic HPV types can lead to a range of cancers, including cervical, anal, penile, and oropharyngeal malignancies [3]. Because HPV transmission frequently occurs without visible symptoms, particularly among sexually active young adults, prevention strategies relying solely on individual risk perception remain insufficient [4].

HPV prevention efforts have focused predominantly on women, largely due to the strong association between HPV and cervical cancer and the availability of organized cervical screening programs [5,6]. Consequently, research priorities, vaccination policies, and public health messaging have disproportionately targeted female populations [7,8]. However, a growing body of evidence challenges this female-centric paradigm, demonstrating that men play a crucial role in HPV transmission dynamics and bear a substantial burden of HPV-related diseases [8,9]. This has prompted increasing debate regarding the effectiveness and equity of female-only vaccination strategies, with some scholars advocating for gender-neutral vaccination policies, while others argue that herd immunity achieved through female vaccination alone may be sufficient in resource-constrained settings [10].

Young adults, particularly university students, represent a population with heightened vulnerability to HPV infection. Individuals aged 18–24 are more likely to engage in behaviors associated with increased HPV exposure, including multiple sexual partnerships and inconsistent condom use [11]. At the same time, studies have reported limited HPV-related knowledge, low perceived susceptibility, and misconceptions regarding vaccine eligibility among young men, which may impede informed decision-making and vaccine uptake [12]. While existing research has examined associations between HPV knowledge, attitudes, and vaccination outcomes, much of this evidence originates from Western countries or mixed-gender samples, raising concerns about its generalizability to socioculturally distinct contexts such as Southeast Asia [13].

In Malaysia, HPV vaccination was introduced into the National Immunisation Programme in 2011 but remains restricted to adolescent girls, effectively excluding males from routine public-sector vaccination coverage [14]. As a result, HPV vaccination among men largely depends on self-financed access, personal health awareness, and individual decision-making processes [15]. Within this female-focused vaccination framework, awareness of HPV vaccination may function as a critical intermediate cognitive determinant, linking knowledge acquisition to actual vaccination behavior [16]. This awareness is shaped not only by individual knowledge but also by sociocultural norms, informational accessibility, and institutional signals regarding vaccine relevance and eligibility. Despite its potential importance, empirical evidence on HPV-related knowledge and vaccination awareness among Malaysian male university students remains scarce, as local studies have primarily focused on women or healthcare-associated populations [17,18,19].

Against this background, the present study aims to assess HPV-related knowledge and awareness of HPV vaccination among male university students in Selangor, Malaysia, and to examine their associations with sociodemographic, behavioral, and cognitive factors. By conceptualizing vaccine awareness as an intermediate cognitive stage within a male-excluded vaccination system, this study seeks to extend beyond traditional descriptive knowledge–attitude–practice (KAP) frameworks. The findings are expected to provide context-specific evidence to inform male-inclusive HPV prevention strategies and contribute to ongoing policy discussions regarding gender-neutral HPV vaccination in Southeast Asia.

## 2. Materials and Methods

### 2.1. Study Design and Participants

A cross-sectional study was conducted among male students from one public and one private university in Selangor, Malaysia. Eligible participants were full-time male students aged 18 years or older who were able to read and understand English. Students who were female, below 18 years of age, or not enrolled as full-time students were excluded.

Due to institutional restrictions on access to official student registries, a convenience sampling approach was employed. Participants were recruited using a QR code that directed them to an anonymous, online, self-administered questionnaire hosted on Google Forms. The QR code was disseminated through university communication channels and student networks, including faculty announcements, student WhatsApp and Telegram groups, and notice boards. Participation was entirely voluntary, and no financial or academic incentives were provided.

A total of 335 questionnaires were distributed. After excluding incomplete or invalid responses (defined as questionnaires with substantial missing data or patterned responses), 302 responses were retained for final analysis, yielding a response rate of 90.1%.

Ethical approval for the study was obtained from the Research Involving Human Subjects Committee of Universiti Putra Malaysia (JKEUPM-2024-199). Electronic informed consent was obtained from all participants prior to questionnaire completion.

### 2.2. Questionnaire

The questionnaire was adapted from previously validated instruments [20,21,22] and consisted of six sections: sociodemographic characteristics (including age, ethnicity, religion, academic level, field of study, and monthly income or allowance); sexual behavior (including history of sexual intercourse); knowledge of HPV and HPV vaccination (covering modes of transmission, health consequences, and vaccine-related information); awareness of HPV vaccination; attitudes toward HPV vaccination; and willingness to receive the HPV vaccine. Awareness of HPV vaccination was operationalized as a binary variable for the primary analysis. The internal consistency of the questionnaire was assessed using Cronbach’s alpha, which demonstrated good reliability, with an overall coefficient of 0.884.

### 2.3. Statistical Analysis

Data were analyzed using SPSS version 26.0. Descriptive statistics were used to summarize participant characteristics and study variables. Categorical variables were presented as frequencies and percentages.

Bivariate associations between independent variables and HPV vaccination awareness were examined using chi-square tests. Variables with a *p*-value < 0.20 in bivariate analyses were considered for inclusion in multivariable logistic regression models. Multivariable logistic regression was conducted to identify factors independently associated with HPV vaccination awareness, and adjusted odds ratios (AORs) with 95% confidence intervals (CIs) were reported.

A *p*-value < 0.05 was considered statistically significant. In addition to the primary analysis using dichotomized knowledge and awareness measures, sensitivity analyses were performed by treating the HPV knowledge score as a continuous variable to assess the robustness of observed associations.

## 3. Results

### 3.1. Demographic Characteristics and Sexual Intercourse

A total of 335 questionnaires were distributed to male students from one public and one private university in Selangor, of which 302 were deemed valid, yielding a response rate of 90.1%. Among the respondents, 55.6% (*n* = 168) demonstrated poor knowledge, while 44.4% (*n* = 134) demonstrated good knowledge regarding HPV, HPV infection, and HPV vaccination. With respect to awareness, 66.2% (*n* = 200) reported having heard of HPV, whereas 33.8% (*n* = 102) had not. Similarly, 55.0% (*n* = 166) were reported being aware of the HPV vaccine, while 45.0% (*n* = 136) reported no prior awareness.

Table 1 summarizes the demographic characteristics of the participants. The majority of respondents were aged 18–24 years (54.6%). Malays (37.7%) and Chinese (44.0%) constituted the largest ethnic groups, and nearly half of the participants identified as Muslim (45.0%). Slightly more than half of the respondents were enrolled in public universities (55.6%), while 44.4% attended private institutions, with over two-thirds pursuing studies in the social sciences. Regarding relationship status, 54.6% of participants were single, and 45.4% were in a relationship. In addition, 44.0% of respondents reported a history of sexual intercourse, and 2.0% reported same-sex sexual intercourse.

### 3.2. Awareness of HPV Vaccination Among Male University Students

**Figure 1:**
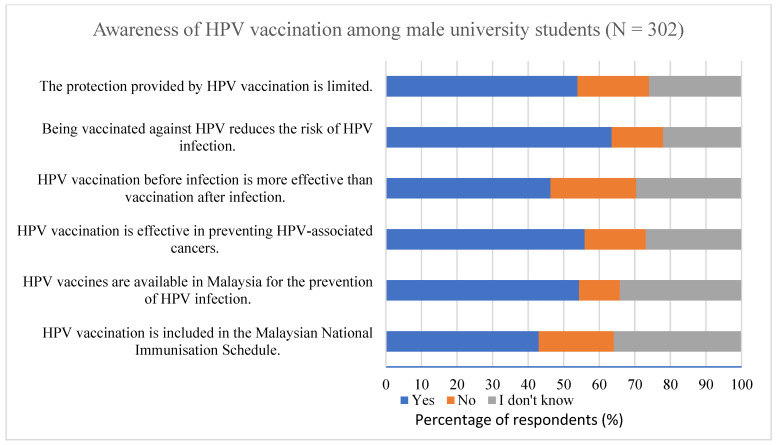
Awareness of HPV vaccination among male university students (N = 302).

Figure 1 shows the respondents’ awareness of HPV vaccination. Less than half of the respondents (43.0%) were aware that the HPV vaccine was included in the Malaysian National Immunisation Programme. Slightly more than half (54.3%) recognized that HPV vaccination reduces the risk of HPV infection; however, a substantial proportion remained uncertain about the vaccine’s specific preventive effects. Regarding cancer prevention, 55.9% of respondents acknowledged that HPV vaccination is effective in preventing HPV-associated cancers, while 44.1% either disagreed or reported not knowing. In addition, fewer than half of the respondents (46.3%) were aware that vaccination before HPV infection is more effective than after infection, indicating limited awareness of the optimal timing of HPV vaccination.

### 3.3. Knowledge Level of the Respondents in Key Items

Table 2 presents respondents’ levels of knowledge across key HPV-related items, illustrating the proportions who answered “yes,” “no,” or “I don’t know” for each question. Overall, 66.8% of respondents correctly identified HPV as a sexually transmitted infection; however, nearly one-third lacked accurate knowledge regarding its transmission. Only 54.3% were aware that condom use can reduce the risk of HPV infection, while almost half of the respondents were uncertain about this preventive measure.

Knowledge regarding gender susceptibility was limited, with only 49.3% recognizing that HPV affects both men and women equally; 28.1% believed otherwise, and 22.5% reported not knowing. Similarly, 55.2% acknowledged that an HPV vaccine exists and can prevent HPV infection, whereas the remaining respondents lacked this awareness, indicating gaps in understanding of vaccination benefits. Notably, only 32.4% correctly identified that antibiotics are not an effective treatment for HPV infection, suggesting a prevalent misconception regarding HPV management. In addition, 54.9% were aware that HPV infection is often asymptomatic, leaving a substantial proportion uncertain about this characteristic, which may influence risk perception and engagement in preventive behaviors.

### 3.4. Univariate Analysis for the Awareness of HPV Vaccination Among Male University Students

Table 3 summarizes the associations between various factors and HPV vaccination awareness. Age was not significantly associated with vaccine awareness, with similar levels observed across age groups; the highest proportion of awareness was reported among respondents aged 18–24 years (65.7%). A significant association was observed for race, with Chinese respondents demonstrating higher awareness (54.2%) compared with Malay (35.9%) and Indian respondents (7.6%). Religious affiliation was also significantly associated with awareness, as non-Muslim respondents showed higher awareness (64.9%) than Muslim respondents (35.1%). Relationship status was significantly related to awareness, with single respondents exhibiting lower awareness (33.6%) than respondents who were in a relationship or married (66.4%). University type was another differentiating factor, with respondents from public universities reporting higher awareness than those from private universities. In contrast, faculty type was not significantly associated with HPV vaccination awareness. History of sexual intercourse was significantly associated with awareness, as respondents who reported having had sexual intercourse demonstrated slightly higher awareness (51.2%) than those without such experience (48.8%). Additionally, HPV-related knowledge level was strongly associated with awareness: respondents with higher HPV-related knowledge were substantially more likely to report awareness of HPV vaccination (71.8%) than those with lower knowledge levels (28.3%).

### 3.5. Logistic Regression with Multiple Covariates for the Awareness of HPV Vaccination Among Male University Students

As shown in Table 4, HPV vaccination awareness was dichotomized into two catego ries (good vs. poor) based on the median score to facilitate interpretability and comparability with prior KAP studies, with poor awareness used as the reference category. Variables with *p* < 0.20 in the univariate analyses were entered into the multivariable logistic regression model, along with age, race, religion, relationship status, history of sexual intercourse, and HPV-related knowledge level. In the adjusted model, non-Muslim respondents were significantly more likely to report good awareness of HPV vaccination compared with Muslim respondents (AOR = 2.724, 95% CI: 1.150–6.454, *p* < 0.001). Similarly, respondents who were in a relationship or married demonstrated higher awareness than single respondents, with a 3.83-fold higher likelihood of reporting good awareness (AOR = 3.830, 95% CI: 2.071–7.082, *p* < 0.001). HPV-related knowledge level showed the strongest association with HPV vaccination awareness, as respondents with good HPV-related knowledge were approximately seven times more likely to report good awareness compared with those with poor knowledge (AOR = 7.012, 95% CI: 4.077–12.059, *p* < 0.001). In contrast, history of sexual intercourse was not significantly associated with HPV vaccination awareness in the adjusted analysis (*p* = 0.097). Sensitivity analyses using the continuous knowledge score yielded results consistent with those of the primary dichotomized model, demonstrating similar directions and levels of statistical significance.

## 4. Discussion

This study examined HPV-related knowledge and awareness of HPV vaccination among male university students in Selangor, Malaysia, a population situated within a vaccination system that remains largely female-focused [21]. Within this policy context, male students may encounter fewer institutional cues that frame HPV vaccination as personally relevant [23]. Accordingly, awareness should not be understood merely as a descriptive outcome, but rather as an intermediate cognitive stage through which exposure to information is translated into vaccine-related decision-making.

Approximately 55% of respondents reported having heard of HPV, a level higher than that reported among male university students in Beijing but lower than that observed in countries such as the United States and Australia, where male HPV awareness often exceeds 70% [24,25]. These differences likely reflect variation in vaccination policies and health communication strategies, especially the earlier and gender-inclusive implementation of HPV vaccination programs in high-income settings [26,27]. However, given the voluntary nature of participation and recruitment from only two universities, respondents may represent a more health-conscious or information-exposed subgroup, suggesting that the observed awareness level may constitute an upper-bound estimate rather than a population-representative prevalence among Malaysian male university students.

In Malaysia, HPV vaccination is included in the National Immunisation Programme exclusively for adolescent girls, leaving males reliant on self-paid vaccination and individual health-seeking behavior [28,29]. Within this institutional context, awareness formation among men may be shaped more strongly by informational environments, such as exposure to health campaigns, university-based messaging, peer networks, and digital health information. This interpretation is consistent with the finding that awareness remains modest despite moderate levels of general HPV knowledge, even among a potentially more health-engaged sample, highlighting a demand-side gap in male vaccination systems [30].

Substantial gaps were identified in respondents’ understanding of HPV infection and prevention. While most participants recognized HPV as a sexually transmitted infection, fewer understood that HPV affects men and women equally, is frequently asymptomatic, or cannot be treated with antibiotics. These findings mirror international evidence indicating a persistent global shortfall in male-oriented HPV education [31]. Misconceptions regarding condom effectiveness were also common; although condoms reduce the risk of HPV transmission, they do not provide complete protection, and overreliance on this belief may lower perceived susceptibility and weaken the perceived need for vaccination [32]. From a behavioral decision-stage perspective, such misconceptions may hinder progression from knowledge acquisition to meaningful awareness by limiting recognition of personal risk [33].

Several sociodemographic and cognitive factors—including race, religion, relationship status, and knowledge level—were associated with HPV vaccination awareness. In a multicultural and multireligious setting such as Malaysia, awareness formation is likely shaped by differential access to health information, variations in social networks, institutional exposure, and health communication pathways, rather than by demographic identity perse [34,35]. Moreover, the magnitude of these associations may have been influenced by the sampling strategy, as patterns observed within a self-selected university sample may not fully generalize to male students with lower levels of health engagement or more limited access to health information.

The strong association between HPV-related knowledge and vaccination awareness reinforces the conceptual distinction between these constructs. While knowledge provides the informational foundation, awareness represents a modifiable gateway through which knowledge is translated into engagement with vaccination decisions [36,37]. This perspective extends beyond traditional KAP models by highlighting a critical gap between knowledge provision and vaccine demand generation. From a vaccination science and implementation perspective, interventions targeting awareness, perceived relevance, and contextual cues may therefore be more effective than knowledge-based strategies alone in improving HPV vaccine uptake among male populations historically excluded from routine immunization programs [38,39].

Barriers to HPV vaccination identified in this study—including embarrassment related to sexual transmission and concerns about vaccine cost—illustrate how individual perceptions interact with broader structural constraints. In conservative sociocultural contexts, HPV vaccination may be implicitly associated with sexual activity, generating discomfort or stigma that inhibits engagement, while financial barriers are particularly salient in settings where male vaccination is not publicly subsidized.

Several limitations should be acknowledged. Convenience sampling from only two universities may have introduced selection bias, potentially leading to an overestimation of HPV-related awareness, knowledge, and positive attitudes. In addition, dichotomization of knowledge and awareness scores may have reduced variability and statistical power; however, sensitivity analyses using continuous measures supported the robustness of the main findings. Findings related to same-sex sexual intercourse should be interpreted cautiously and were considered exploratory due to the small subgroup size.

Overall, this study advances HPV vaccination research by situating male awareness within broader vaccination decision-making and implementation frameworks. By linking descriptive findings to behavioral mechanisms, demand-side barriers, and institutional contexts, the study provides policy-relevant insights for male-inclusive HPV vaccination strategies in university-based settings and informs future longitudinal and intervention-based research in similar sociocultural contexts.

## 5. Conclusions

This study demonstrates that HPV vaccination awareness among male university students in Malaysia remains limited and is largely influenced by knowledge and contextual factors within a predominantly female-focused vaccination framework. Awareness is conceptualized as a key cognitive link between knowledge and vaccine-related decision-making. These findings suggest that improving knowledge alone is insufficient unless combined with strategies that explicitly enhance awareness and address structural and communication barriers. Male-inclusive, university-based, and data-driven interventions may therefore be essential for improving HPV vaccine engagement among young men. Further longitudinal and intervention-based studies are needed to validate these pathways and inform vaccination strategies in similar sociocultural contexts.

## Figures and Tables

**Table 1:** Demographic characteristics and sexual intercourse of the participants (N = 302).

Variable	Categories	*n* (%)
Age (years)	18–24	165 (54.6)
	25–32	101 (33.4)
	>33	36 (11.90)
Race	Malay	114 (37.7)
	Chinese	133 (44)
	Indian	38 (9.3)
	Other	27 (8.9)
Religion	Muslim	136 (45)
	Non-Muslim	166 (55)
University Type	Private University	134 (44.4)
	Public University	168 (55.6)
Faculty	Science	100 (33.1)
	Social Science	202 (66.9)
Relationship Status	Single	165 (54.6)
	Non-single	137 (45.4)
Monthly household income (MYR)	<2000	81 (26.8)
	2000–3999	84 (27.8)
	4000–6000	67 (22.2)
	>6000	70 (23.2)
History of sexual intercourse	Yes	133 (44)
	No	169 (56)
History of same-sex intercourse	Yes	6 (2.0)
	No	296 (98)

**Table 2:** Level of knowledge regarding HPV among male university students (N = 302).

Knowledge Items	Yes, *n* (%)	No, *n* (%)	I Don’t Know, *n* (%)
HPV is a type of sexually transmitted disease.	202 (66.8)	36 (11.9)	64 (21.1)
HPV does infect males and females equally.	149 (49.3)	85 (28.1)	69 (22.5)
HPV infection is common in Malaysia.	81 (26.8)	98 (32.4)	123 (40.7)
Antibiotics are not an effective treatment for HPV infection.	98 (32.4)	70 (23.1)	134 (44.3)
People who are infected do not have visible signs or symptoms.	166 (54.9)	55 (18.2)	81 (26.8)
HPV can be prevented by using a condom during sexual intercourse.	164 (54.3)	47 (15.5)	91 (30.1)
A vaccine against HPV infection does exist.	167 (55.2)	36 (11.9)	99 (32.7)

**Table 3:** Univariate analysis of factors associated with awareness of HPV vaccination among male university students (N = 302).

Variable	Categories	Level of Awareness		χ^2^	*p*-Value
		Poor, *n* (%)	Good, *n* (%)		
Age (years)	18–24	97 (65.7)	68 (51.9)	3.877	0.144
	23–32	50 (29.2)	51 (38.9)		
	>33	24 (14.0)	12 (9.2)		
Race	Malay	67 (39.2)	47 (35.9)	17.750	<0.001
	Chinese	62 (36.3)	71 (54.2)		
	Indian	18 (10.5)	10 (7.6)		
	Other	24 (14.0)	3 (2.3)		
Religion	Muslim	90 (52.6)	46 (35.1)	9.195	0.002
	Non-Muslim	81 (47.4)	85 (64.9)		
University Type	Private University	67 (39.2)	67 (51.1)	4.301	0.038
	Public University	104 (60.8)	64 (48.9)		
Faculty	Science	54 (31.6)	46 (35.1)	0.419	0.518
	Social Science	117 (68.4)	85 (64.9)		
Relationship Status	Single	121 (70.8)	44 (33.6)	41.353	<0.001
	Non-Single	50 (29.2)	87 (66.4)		
Monthly household income (MYR)	<2000	47 (27.5)	34 (26.0)	2.322	0.508
	2000–3999	52 (30.4)	32 (24.4)		
	4000–6000	37 (21.6)	30 (22.9)		
	>6000	35 (20.5)	35 (26.7)		
History of sexual intercourse	Yes	66 (38.6)	67 (51.2)	4.739	0.029
	No	105 (61.4)	64 (48.8)		
History of same-sex intercourse	Yes	1 (0.6)	5 (3.8)	3.979	0.046
	No	170 (99.4)	126 (96.2)		
HPV-related knowledge level	Poor	131 (76.6)	37 (28.3)	70.292	<0.001
	Good	40 (23.4)	94 (71.8)		

**Table 4:** Multivariable logistic regression analysis of factors associated with awareness of HPV vaccination among male university students (N = 302).

Variable	Categories	AOR	95% CI	*p*-Value
Religion	Muslim	1.00		
	Non-Muslim	2.724	1.150–6.451	<0.001
Relationship	Single	1.00		
	Non-Single	3.830	2.071–7.082	<0.001
History of Sexual intercourse	No	1.00		
	Yes	0.584	0.310–1.102	0.097
HPV-related Knowledge level	Poor	1.00		
	Good	7.012	4.077–12.059	<0.001

## Data Availability

The data that support the findings of this study are available from the corresponding author upon reasonable request.

## Abbreviations

The following abbreviations are used in this manuscript:

HPV	Human papillomavirus
KAP	Knowledge–attitude–practice
